# Characteristics and outcomes of NATALEE- and monarchE-eligible early breast cancer in Finland: a regional real-world cohort study

**DOI:** 10.1093/oncolo/oyag419

**Published:** 2026-10-20

**Authors:** Sari Voutilainen, Samuli Tuominen, Liisa Ukkola-Vuoti, Sari Hernesniemi, Mia Engström-Risku, Björn Paulsson, Maija Wolf, Peeter Karihtala

**Affiliations:** Department of Oncology, Helsinki University Hospital Comprehensive Cancer Center, University of Helsinki, Helsinki FI-00029, Finland; Medaffcon Oy, Espoo, 02150, Finland; Medaffcon Oy, Espoo, 02150, Finland; Novartis Finland Oy, Espoo, 02100, Finland; Novartis Finland Oy, Espoo, 02100, Finland; Novartis Sverige AB, Stockholm, 16440, Sweden; Novartis Finland Oy, Espoo, 02100, Finland; Department of Oncology, Helsinki University Hospital Comprehensive Cancer Center, University of Helsinki, Helsinki FI-00029, Finland

**Keywords:** HR+, HER2−, early breast cancer, RWE, CDK4/6 inhibitors, recurrence risk

## Abstract

**Background:**

Adjuvant cyclin-dependent kinases 4 and 6 (CDK4/6) inhibitors have improved outcomes in hormone receptor-positive/human epidermal growth factor receptor 2-negative (HR+/HER2–) early breast cancer (eBC) in the NATALEE and monarchE trials. However, real‑world evidence on the proportion of candidates for adjuvant CDK4/6 inhibitors and their outcomes within these trial-defined populations remains limited.

**Materials and Methods:**

We conducted a retrospective, population‑based cohort study including all patients diagnosed with HR+/HER2– eBC in the Hospital District of Helsinki and Uusimaa, Finland, between January 2014 and June 2023. Patients were classified according to NATALEE and/or monarchE eligibility criteria. Outcomes assessed were invasive disease‑free survival (iDFS), distant disease‑free survival (DDFS), and overall survival (OS).

**Results:**

Among 8911 patients with HR+/HER2- eBC and a median follow-up of 5.2 years, 40.4% met the NATALEE eligibility criteria and 15.0% met the monarchE criteria. Eligible patients were younger and had higher‑grade tumors and greater proliferation rate than non-eligible patients. Five‑year outcomes were consistently worse in eligible than ineligible patients (NATALEE: iDFS 82.5% vs 89.6%, DDFS 88.3% vs 94.5%, OS 90.9% vs 95.6%; monarchE: iDFS 76.8% vs 88.4%, DDFS 82.3% vs 93.8%, OS 86.8% vs 94.9%). Metastatic progression occurred 3‑ to 6‑fold more often in eligible patients. In multivariable models, both NATALEE and monarchE eligibility were associated with distant metastasis, and mortality.

**Conclusion:**

Real-world outcomes among NATALEE and monarchE-eligible patients are consistent with results from phase III trial comparator arms. Trial‑defined eligibility criteria reliably identify patients with substantially poorer prognosis, supporting risk‑adapted adjuvant strategies and the integration of CDK4/6 inhibitors into clinical practice.

Implications for practice A substantial proportion of real‑world patients with HR+/HER2− early breast cancer meet the NATALEE or monarchE eligibility criteria and show markedly poorer long‑term outcomes than ineligible patients when treated according to pre-CDK4/6 standard of care. These trial‑based criteria define a prognostically distinct high-risk subgroup in routine practice, including high‑risk node‑negative patients. Our population-based data provides robust estimates of the frequency, characteristics, and baseline prognosis of trial-eligible patients, offering essential context for benefit-risk assessment and for evaluating the potential population-level impact of implementing adjuvant CDK4/6 inhibition in clinical practice.

## Introduction

Breast cancer (BC) is the most common cancer and the leading cause of cancer-related mortality among women worldwide.^1^ In 2022, approximately 2.3 million women were diagnosed with BC globally, and the mortality rate of BC was 12.7 per 100 000 women.^2^ In Finland during the same year, 4870 women and 30 men were diagnosed with BC, with mortality rates of 31.0 per 100 000 and 0.18 per 100 000, respectively.^3^ Approximately 70% of early breast cancer (eBC) cases are hormone receptor-positive and human epidermal growth factor receptor 2-negative (HR+/HER2–).^4^^,^^5^ Due to effective screening programs, an increasing proportion of BC cases are now detected at an early stage.^6^

Management of HR+/HER2- eBC entails a personalized, multidisciplinary approach that typically includes surgery, radiotherapy, systemic therapies—chemotherapy (CT) and endocrine therapy (ET)—and targeted interventions.^7^ Cyclin-dependent kinases 4 and 6 (CDK4/6) are key regulators of cell-cycle progression and play an important role in the initiation, growth, and survival of many cancers. Inhibition of CDK4/6 induces tumor-cell senescence and modulates antitumor immunity by promoting cytotoxic T-cell-mediated tumor clearance.^8^^,^^9^ CDK4/6 inhibitors have rapidly become a standard of care in advanced HR+/HER2– BC,^9^ and their use has since been broadened to the adjuvant setting. Despite standard adjuvant endocrine therapy (ET) with or without chemotherapy, a substantial proportion of patients with HR+/HER2– eBC experience disease recurrence, with recurrence risk largely determined by clinicopathologic features, such as nodal involvement, proliferation index, and tumor size.^10^^,^^11^

The phase III NATALEE^10^^,^^12^^,^^13^ and monarchE^14–17^ trials have demonstrated that adjuvant CDK4/6 inhibition reduces the risk of recurrence (RoR) in patients with HR+/HER2– eBC, leading to regulatory approvals of ribociclib and abemaciclib by the European Medicines Agency (EMA) and the United States Food and Drug Administration (FDA). In monarchE, 2 years of abemaciclib added to ET resulted in a sustained long-term benefit.^17^ At 7 years, iDFS was 77.4% with abemaciclib plus ET vs 70.9% with ET alone (absolute difference 6.5%), corresponding to a hazard ratio (HR) of 0.734 (95% CI 0.657-0.820).^17^ Importantly, a statistically significant OS benefit was observed, with a 7-year OS of 86.8% vs 85.0% (absolute difference 1.8%; HR 0.842, 95% CI 0.722-0.981; *P* = .027).^17^ In NATALEE, 3 years of ribociclib combined with ET significantly reduced the risk of invasive recurrence.^18^ At 5 years, iDFS remained improved between treatment arms, with an absolute difference of 4.5% (HR 0.716, 95% CI 0.618-0.829).^18^ OS data from NATALEE remain immature, although a favorable numerical trend has been reported (HR 0.800, 95% CI 0.637-1.003).^18^ These findings established adjuvant CDK4/6 inhibitors as a treatment option for selected patients at high risk of recurrence. However, the real-world applicability of trial eligibility criteria and the long-term outcomes of patients meeting these criteria outside clinical trial settings remain to be characterized. In particular, population-based data are needed to quantify the proportion of patients who would qualify for adjuvant CDK4/6 inhibition and define their baseline prognosis in routine care. Such information is essential for contextualizing the potential clinical impact of trial-based treatment strategies and for informing their implementation in unselected patient populations. Several recent Nordic real-world studies have also examined outcomes in patients with high-risk HR+/HER2– early breast cancer.^19–21^ A Danish population-based study reported recurrence risks and long-term outcomes broadly consistent with those observed in CDK4/6 inhibitor trial populations,^21^ while 2 large Swedish registry analyses—one evaluating patients eligible for adjuvant abemaciclib or ribociclib^19^ and another assessing recurrence and survival in stage II-III ER+/HER2– disease reflecting the NATALEE inclusion criteria in terms of anatomical stage^20^—have similarly highlighted the substantial residual risk in these trial-defined populations. These findings underscore the importance of region-specific real-world data to contextualize treatment needs in Nordic healthcare settings.

The aim of this study was to evaluate the real‑world proportion of eBC patients who meet eligibility criteria for adjuvant CDK4/6 inhibitors and to characterize the clinicopathologic features, treatments, and long-term outcomes within the Hospital District of Helsinki and Uusimaa (HUS), Finland.

## Methods

### Study design and data source

This retrospective, non-interventional cohort study used routinely collected electronic health record data from the HUS data lake, which contains comprehensive secondary care information from 23 hospitals serving approximately 1.6 million residents in the Helsinki and Uusimaa region. Finland has a tax-funded, universally accessible healthcare system, in which cancer diagnostics and treatment are largely centralized within regional hospital districts. This structure enables comprehensive capture and longitudinal follow-up of all patients diagnosed with HR+/HER2– eBC in the HUS area, supporting the population-based design and minimizing selection bias. The study was conducted under the data permit *HUS/1364/2024* in accordance with the Finnish Act on the Secondary Use of Health and Social Data (552/2019). Pseudonymized data were used for the analysis within the secure HUS Acamedic analysis environment; therefore, informed consent was not required. The study complied with Finnish data protection legislation. Data remained pseudonymized at all stages, and no individual-level data were accessible outside the secure analysis platform. No patient contact occurred. The study adhered to the STrengthening the Reporting of OBservational studies in Epidemiology (STROBE) reporting guideline.^22^

Available data included diagnoses, procedures, hospital-administered and prescribed medications, structural pathology reports, and laboratory results. Relevant information was also extracted from radiology reports and free-text clinical notes containing references to metastasis. Pathology data were available from 2013 onward, while data on diagnoses, treatments, and laboratory results were available from 2010 onward. Background medical history (2010-2013) was used to capture baseline comorbidities.

### Study population

The source population comprised all adult patients (≥18 years at diagnosis) diagnosed with breast cancer (ICD10: C50) in the HUS region between 2010 and 2023, corresponding to the period with complete and reliable diagnostic coverage up to the most recent data available at the time of extraction. From this population, the study cohort included patients with a first diagnosis of invasive stage I-III HR+/HER2− BC between January 1, 2014 and June 30, 2023, according to the AJCC 8th Edition anatomical staging system,^23^ allowing for a 4-year washout period to detect patients whose date of diagnosis was before 2014, but whose follow-up contacts occurred during the study period. Patients detected with distant metastasis during adjuvant chemotherapy or radiotherapy, or within 3 months of diagnosis (to allow for at least 1 radiological assessment), were excluded, as well as those with any prior breast cancer diagnosis during the washout period before 2014 (prevalent cases). Patients with documented CDK4/6 inhibitor use prior to reimbursement of these agents for advanced breast cancer were excluded. Use of adjuvant CDK4/6 inhibitors was negligible during the study period because these treatments were not routinely available in Finnish public healthcare and Helsinki University Hospital did not participate in the monarchE trial. Patients were followed from the date of BC diagnosis until the development of advanced breast cancer, death, or end of follow-up (June 30, 2024), whichever occurred first. All patients had a minimum follow-up of 12 months.

### Biological subtype, distant metastasis, and variable definitions

HR positivity was defined as ≥1% of tumor cells showing positive nuclear staining for estrogen or progesterone, or if a patient received ET. HER2 negativity was determined from pathology records as a negative *in situ* hybridization (ISH) result or an immunohistochemistry (IHC) score of 0 or 1+, when ISH results were unavailable. In case of multiple ISH/IHC records all results were required to be negative. Patients who received anti-HER2 treatment were excluded irrespective of recorded HER2 status.

Advanced breast cancer (aBC) was defined based on records indicative of distant metastasis. Distant metastases were identified using a composite definition incorporating mentions of metastasis in free-text clinical notes, the International Classification of Diseases, 10th revision (ICD-10) diagnosis codes (C78 or C79), pathologist affirmed distant metastasis on biopsy, documentation of TxNxM1 or Stage IV disease in clinical notes, use of systemic therapies specific to HR+/HER2- aBC (including bevacizumab, carboplatin, cisplatin, eribuline, gemcitabine, vinorelbine, olaparib, talazoparib, methotrexate, and denosumab with 120 mg dose), aBC mentioned in the prescription label, and procedures indicative of treatment for metastatic disease. The earliest recorded date from these sources was considered the date of distant metastasis or onset of aBC. Cases with prior malignancy or atypical first-line treatment patterns (such as fulvestrant or chemotherapy) were manually reviewed using radiological and clinical records to confirm distant metastasis originating from breast cancer.

Tumor characteristics, including histological subtype, grade, Ki67 expression, tumor size (T), and nodal status (N), were derived from pathology reports and structured electronic medical records. Menopausal status was defined as age ≥60 years, prior bilateral oophorectomy, use of aromatase inhibitor without ovarian suppression, or laboratory criteria (FSH ≥26.7 U/L and estradiol <0.10 nmol/L); otherwise, patients were considered as premenopausal. Comorbidity burden was assessed using the Charlson Comorbidity Index (CCI).^24^

### NATALEE and monarchE eligibility criteria

Patients were classified according to the published pathological and anatomical eligibility criteria applied in the NATALEE and monarchE (Cohort 1) trials (Table S1). Patients were defined as NATALEE‑eligible^13^ if they had HR+, HER2- eBC and presented with anatomical stage IIA with N1; or stage IIA with N0 and either grade 3 disease or grade 2 disease with a Ki‑67 score ≥20% (genomic risk could not be assessed as an additional risk factor). In addition, patients with anatomical stage IIB (N0 or N1) and patients with anatomical stage III were also considered eligible. Patients were defined as monarchE eligible (Cohort 1)^12^ if they had HR+/HER2- node-positive eBC with either ≥ 4 positive axillary lymph nodes (ALN), or 1 to 3 positive ALNs combined with tumor size ≥ 5 cm and/or grade 3 disease. Patients who did not fulfill eligibility criteria for NATALEE or monarchE, were classified as NATALEE or monarchE ineligible, respectively.

### Outcomes

Three clinical outcomes were assessed: overall survival (OS), distant disease-free survival (DDFS), and invasive disease-free survival (iDFS). OS was defined as time from BC diagnosis to death from any cause. DDFS was defined as time from BC diagnosis date to distant metastasis, secondary malignancy or death from any cause. iDFS was defined as time from BC diagnosis date to invasive recurrence (local, regional, or distant), second primary invasive malignancy, or death from any cause. Local recurrence was defined as re-initiation of chemotherapy or radiotherapy more than 90 days after completion of adjuvant chemotherapy (with the earliest accepted date ≥1 year from BC diagnosis date) or a new pathology record of breast cancer >180 days after completion of adjuvant chemotherapy. The definition of iDFS was aligned with the STEEP criteria used in clinical trial settings.^25^^,^^26^ End of follow-up (June 30, 2024) was considered as a censoring date for all outcomes.

### Statistical analysis

Analyses were conducted in R version 4.5.2. Baseline characteristics were summarized using medians and interquartile intervals for continuous variables and number and proportion of patients for categorical variables. Between-group comparisons used Student’s *t* test or χ^2^ test as appropriate. Missing values were not imputed; however, the missing proportions were reported, as applicable. All *P*-values below .05 were considered statistically significant, and 95% CI were used throughout.

iDFS, DDFS, and OS were assessed using the Kaplan–Meier method. Group comparisons were performed using univariable and multivariable Cox proportional hazards models in outcome analyses. To adjust for covariates, multivariable cause-specific Cox models were fitted for iDFS (event defined as recurrence or distant metastasis; patients censored at death, secondary malignancy, or end of follow-up) and DDFS (event defined as distant metastasis; secondary malignancy, patients censored at death or end of follow-up), while standard multivariable Cox models were used for OS. Covariates included age, sex, CCI, year of diagnosis, menopausal status, and histology. The proportional hazards assumptions were checked for all models (data not shown).

### Ethical approval statement

As a retrospective study, no ethical committee evaluation was required under Finnish legislation.

## Results

### Study cohort

A total of 15 054 patients were diagnosed with breast cancer in the HUS region between 2010 and 2023, of whom 8911 had HR+/HER2– eBC. Sufficient clinicopathologic data to evaluate trial eligibility were available for 7806 patients (87.6%) (Figure 1). Among all patients with HR+/HER2– eBC 3596 (40.4%) met the NATALEE eligibility criteria, and 1336 (15.0%) met the monarchE criteria. These proportions were calculated using the entire HR+/HER2- population as denominator, including patients with incomplete data for eligibility assessment; accordingly, 12.4% of all HR+/HER2- patients could not be fully evaluated for trial eligibility. Overall, 4210 patients (47.2%) were classified as NATALEE-ineligible and 6470 (72.6%) as monarchE-ineligible. Of the patients with data available for defining eligibility, 3596 (46.1%) were classified as NATALEE eligible and 1336 (17.1%) monarchE eligible. All monarchE-eligible patients were included within the NATALEE cohort, whereas 2260 patients (62.8% of the NATALEE-eligible cohort) fulfilled NATALEE criteria exclusively. Median follow-up across all HR+/HER2–eBC patients was 5.2 years (interquartile interval, IQI, 3.0-7.7). Follow-up was similar between NATALEE-eligible and ineligible patients (5.4 years for both; *P* = .5), but longer among monarchE-eligible compared with ineligible patients (5.9 vs 5.3 years, *P* < .001) (Table 1).

**Figure 1.**
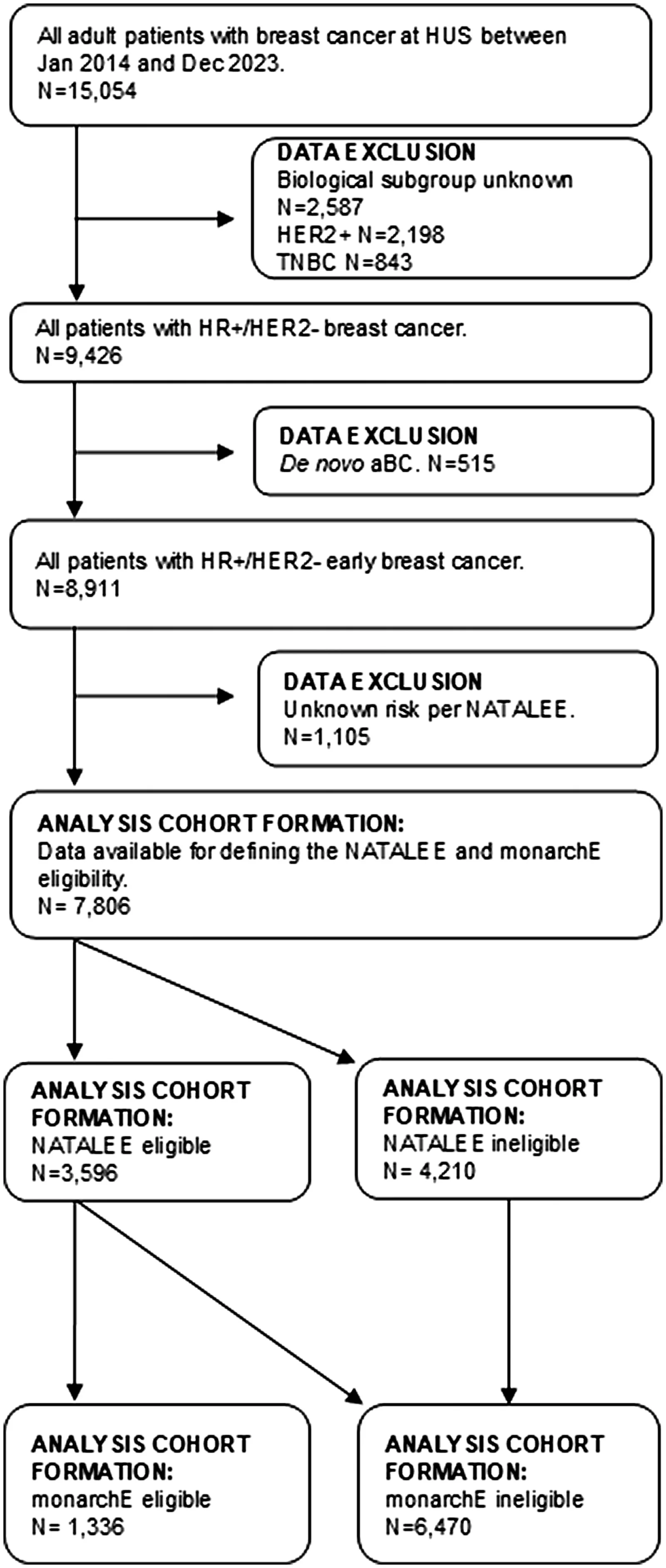
Study design and data flow by NATALEE and monarchE eligibility criteria.

**Table 1.** Baseline characteristics.

Characteristic	All HR+/HER2− eBC patients, *N* = 8911	NATALEE			monarchE		
		Eligible, *N* = 3596	Ineligible, *N* = 4210	*P*-value	Eligible, *N* = 1336	Ineligible, *N* = 6470	*P*-value
**Median age at diagnosis, years (IQI)**	65.7 (56.8, 73.9)	64.8 (54.8, 73.7)	65.5 (58.3, 72.2)	.021	63.7 (53.0, 73.0)	65.3 (57.1, 72.9)	<.001
**Sex**				.023			.004
** Female**	8877 (99.6%)	3576 (99.4%)	4200 (99.8%)		1325 (99.2%)	6451 (99.7%)	
** Male**	34 (0.4%)	20 (0.6%)	10 (0.2%)		11 (0.8%)	19 (0.3%)	
**Postmenopausal^a^**	6723 (75.4%)	2498 (69.5%)	3362 (79.9%)	<.001	847 (63.4%)	5013 (77.5%)	<.001
**Metastatic axillary lymph nodes^b^**	3275 (38.9%)	2981 (82.9%)	0 (0.0%)	<.001	1336 (100%)	1645 (25.4%)	<.001
**Number of metastatic lymph nodes^b^**				<.001			<.001
** 0**	5149 (61.1%)	615 (17.1%)	4210 (100.0%)		0 (0.0%)	4825 (74.6%)	
** 1-3**	2560 (30.4%)	2378 (66.1%)	0 (0.0%)		733 (54.9%)	1645 (25.4%)	
** 4-9**	387 (4.6%)	328 (9.1%)	0 (0.0%)		328 (24.6%)	0 (0.0%)	
** >9**	328 (3.9%)	275 (7.6%)	0 (0.0%)		275 (20.6%)	0 (0.0%)	
**Grade^c^**				<.001			<.001
** 1**	2282 (28.5%)	490 (14.3%)	1645 (41.5%)		66 (5.1%)	2069 (34.0%)	
** 2**	3920 (49.0%)	1739 (50.9%)	1849 (46.6%)		347 (26.7%)	3241 (53.3%)	
** 3**	1805 (22.5%)	1186 (34.7%)	470 (11.9%)		888 (68.3%	768 (12.6%)	
**Tumor size^d^**				<.001			<.001
** 0**	0 (0.0%)	0 (0.0%)	0 (0.0%)		0 (0.0%)	0 (0.0%)	
** ≤2 cm**	5458 (67.9%)	1472 (40.9%)	3830 (91.0%)		449 (33.6%)	4853 (75.0%)	
** 2-5 cm**	2219 (27.6%)	1772 (49.3%)	380 (9.0%)		631 (47.2%)	1521 (23.5%)	
** >5 cm**	353 (4.4%)	346 (9.6%)	0 (0.0%)		253 (18.9%)	93 (1.4%)	
** Spread to surrounding tissue**	6 (0.1%)	6 (0.2%)	0 (0.0%)		3 (0.2%)	3 (0.0%)	
**Ki-67 expression^e^**				<.001			<.001
** Low (<20%)**	4506 (54.1%)	1246 (35.5%)	2907 (71.0%)		266 (20.4%)	3887 (61.7%)	
** High (≥20%)**	3823 (45.9%)	2267 (64.5%)	1186 (29.0%)		1037 (79.6%)	2416 (38.3%)	
**Developed aBC during follow-up**	381 (4.3%)	232 (6.5%)	72 (1.7%)	<.001	166 (12.4%)	138 (2.1%)	<.001
**Charlson Comorbidity Index**				.11			.2
** 0**	7582 (85.1%)	3105 (86.3%)	3647 (86.6%)		1153 (86.3%)	5599 (86.5%)	
** 1**	485 (5.4%)	176 (4.9%)	238 (5.7%)		61 (4.6%)	353 (5.5%)	
** 2 or over**	844 (9.5%)	315 (8.8%)	325 (7.7%)		122 (9.1%)	518 (8.0%)	
**Histology^f^**				<.001			<.001
** Carcinoma ductale**	6107 (71.0%)	2510 (70.7%)	2961 (71.4%)		963 (73.6%)	4508 (70.6%)	
** Carcinoma lobular**	1783 (20.7%)	813 (22.9%)	775 (18.7%)		278 (21.2%)	1310 (20.5%)	
** Carcinoma mucinosum**	188 (2.2%)	44 (1.2%)	116 (2.8%)		8 (0.6%)	152 (2.4%)	
** Carcinoma neuroendocrinum**	29 (0.3%)	16 (0.5%)	9 (0.2%)		< 3	Censored	
** Carcinoma tubulare**	135 (1.6%)	15 (0.4%)	112 (2.7%)		< 3	Censored	
** Multiple histologies**	336 (3.9%)	146 (4.1%)	160 (3.9%)		54 (4.1%)	252 (3.9%)	
** Other^g^**	22 (0.3%)	6 (0.2%)	13 (0.3%)		3 (0.2%)	16 (0.3%)	
**Length of follow-up, years, median (IQI; from BC diagnosis until EOF)**	5.2 (3.0, 7.7)	5.4 (3.1, 7.8)	5.4 (3.3, 7.8)	.5	5.9 (3.4, 8.2)	5.3 (3.1, 7.7)	<.001

Abbreviations: aBC, advanced breast cancer; eBC, early breast cancer; IQI, interquartile interval.

Data are shown as *n* (%) unless otherwise indicated. Due to anonymity guidelines by the Finnish Central Authority Findata, small patient groups (<3 patients) have not been reported in detail. Additionally, numbers in other cells have been censored, if reporting would have allowed inferring the exact number covered by “<3.”

a Men included as premenopausal.

b Missing rate for metastatic lymph nodes among all HR+/HER2− eBC patients was 5.5% (*n* = 487).

c Missing rate for grade among all HR+/HER2− eBC patients was 10.1% (*n* = 328) and subgroups among 2.6%-6.1%.

d Missing rate for tumor size among all HR+/HER2− eBC patients was 9.8% (*n* = 875).

e Missing rate for Ki-67 expression among all HR+/HER2− eBC patients was 6.5% (*N* = 582) and among subgroups 2.5%-2.8%.

f Missing rate for histology among all HR+/HER2− eBC patients was 3.5% (*N* = 311) and among subgroups 1.3%-2.0%.

g Histological subtype one of: ca. apocrinum, ca. metaplasticum, ca. papillare, ca. medullare mammae, or ca. adenomatosum.

### Baseline clinical and pathologic characteristics

The median age at HR+/HER2- eBC diagnosis was 65.7 years (IQI 56.8-73.9; Table 1). Of all patients, 75.8% were postmenopausal. Eligible patients were less frequently postmenopausal than ineligible patients (NATALEE: 69.9% vs 80.0%; monarchE: 64.0% vs 77.7%; *P* < .001; Table 1 and Table S2). Compared with ineligible patients, eligible patients were younger at diagnosis (NATALEE: 64.8 vs 65.5 years, *P* = .021; monarchE: 63.7 vs 65.3 years, *P* < .001). Overall, 34 patients (0.4%) were men.

Histological subtype data were available for 96.5% of patients with eBC, and distributions of histologies differed significantly across eligibility groups (*P* < .001). Tubular carcinoma was more common among NATALEE-ineligible patients (2.7%, *n* = 112) compared with eligible patients (0.4%, *n* = 15). During follow‑up, 4.3% of HR+/HER2– eBC patients developed aBC (Table 1). Progression to aBC was more frequent among eligible compared with ineligible patients (NATALEE: 6.5% vs 1.7%, *P* < .001; monarchE: 12.4% vs 2.1%, *P* < .001).

Among NATALEE-eligible patients, 17.1% were node-negative (N0), and 66.1% were N1, whereas nearly half (45.2%) of monarchE‑eligible patients had N2–3 disease at diagnosis (Table 1; Table S3).

### Treatment patterns

Mastectomy or breast-conserving surgery was performed in 96.9% of HR+/HER2– eBC patients, with similar rates across eligibility groups (Table S4A). ET was the predominant systemic adjuvant treatment, administered to 82.9% of HR+/HER2–eBC patients overall and to 97.3% and 97.8% of NATALEE- and monarchE-eligible patients, respectively. Adjuvant ET was slightly more frequently used in premenopausal than postmenopausal eligible patients, particularly in the monarchE-eligible group (NATALEE: 97.5 vs 97.1; monarchE 99.0% vs 97.1%; Table S4B). Adjuvant chemotherapy was substantially more common among NATALEE-eligible (45.4%) and monarchE-eligible patients (56.0%) compared with their respective ineligible groups (5.3% and 17.2%; Table S4A). Premenopausal patients received adjuvant chemotherapy more often than postmenopausal patients across all strata, particularly in the NATALEE-eligible group (77.3% vs 31.7%; Table S4B).

### Invasive disease–free survival

Across all patients with HR+/HER2– eBC, the 5‑year iDFS was 84.1% (95% CI 83.2-84.9; Figure 2). For NATALEE-eligible patients, 5-year iDFS was 82.5% (95% CI 81.0-83.8) compared with 89.6% (95% CI 88.6-90.6) in NATALEE-ineligible patients (HR 1.63, 95% CI 1.45-1.82, *P* < .001). Corresponding estimates for monarchE were 76.8% (95% CI 74.2-79.1) in eligible and 88.4% (95% CI 87.5-89.2) in ineligible patients (HR 1.93, 95% CI 1.70-2.18, *P* < .001). Kaplan–Meier estimates of iDFS are presented in Figure 2 up to 10 years.

**Figure 2.**
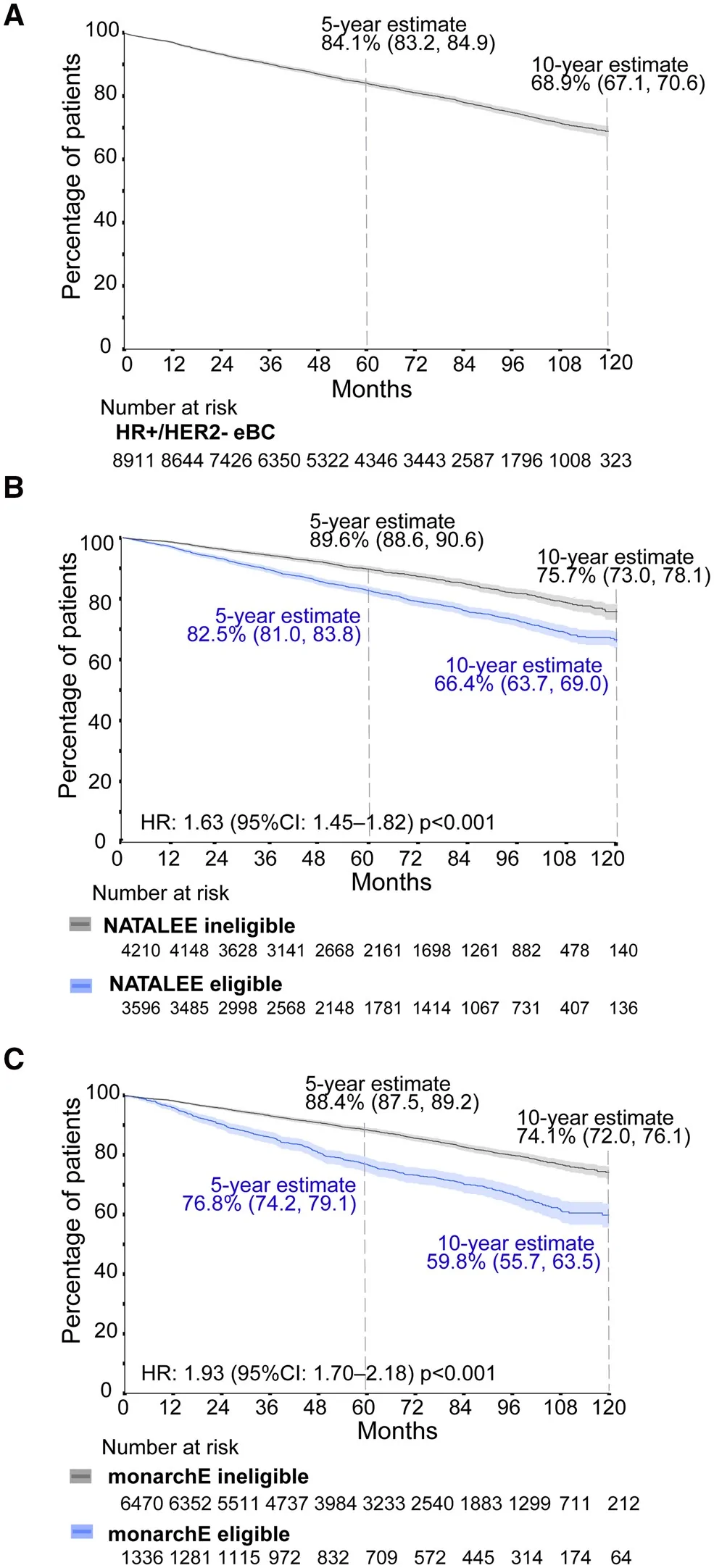
Kaplan-Meier estimates for invasive disease–free survival (iDFS) among patients with HR+/HER2− eBC (A) overall, (B) per NATALEE eligibility criteria (eligible vs ineligible), and (C) per monarchE eligibility criteria (eligible vs ineligible).

In multivariable Cox models, a higher risk of recurrence was associated with eligible NATALEE (HR 1.90; 95% CI 1.58-2.29; *P* < .001) and monarchE (HR 2.64; 95% CI 2.19-3.18; *P* < .001) patients when compared with the corresponding ineligible patients (Figure S1a, see online supplementary material for a color version of this figure).

### Distant disease–free survival

The overall 5‑year DDFS for HR+/HER2– eBC was 89.4% (95% CI 88.7-90.1; Figure 3). For NATALEE‑eligible patients, DDFS was 88.3% (95% CI 87.1-89.5), compared with 94.5% (95% CI 93.7-95.3) in ineligible patients (HR 2.08, 95% CI 1.80-2.41, *P* < .001). Corresponding estimates for monarchE were 82.3% (95% CI 79.9-84.4) in eligible and 93.8% (95% CI 93.0-94.4) in ineligible patients (HR 2.85, 95% CI 2.46-3.29, *P* < .001). Kaplan–Meier estimates of DDFS are shown in Figure 3 up to 10 years.

**Figure 3.**
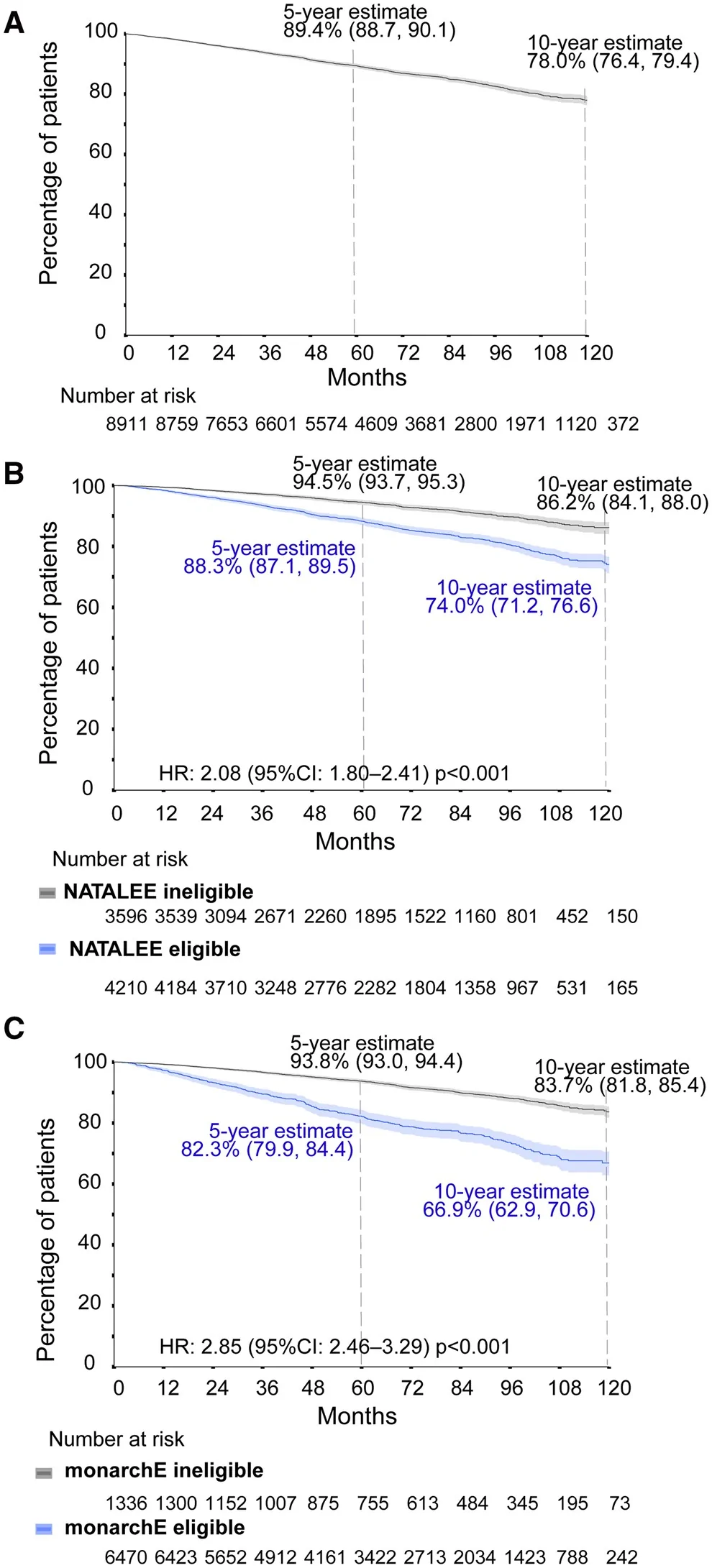
Kaplan-Meier estimates for distant disease–free survival (DDFS) among patients with HR+/HER2− eBC (A) overall, (B) per NATALEE eligibility criteria (eligible vs ineligible), (C) per monarchE eligibility criteria (eligible vs ineligible).

Both NATALEE and monarchE‑eligible patients had a significantly higher risk of distant recurrence compared with ineligible patients (NATALEE-eligible: HR 3.63; 95% CI 2.77-4.76; *P* < .001; monarchE-eligible: HR 5.54; 95% CI 4.39-6.99; *P* < .001; Figure S1b, see online supplementary material for a color version of this figure).

### Overall survival

The 5-year OS across all HR+/HER2– eBC patients was 91.3% (95% CI 90.6-91.9; Figure 4). For NATALEE-eligible patients, 5-year OS was 90.9% (95% CI 89.8-91.9) compared with 95.6% (95% CI 94.8-96.2) in ineligible patients (HR 1.99, 95% CI 1.70-2.32, *P* < .001). For monarchE, corresponding estimates were 86.8% (95% CI 84.4-88.6) and 94.9% (95% CI 88.6-94.3), respectively (HR 2.34, 95% CI 1.99-2.74, *P* < .001). Kaplan–Meier estimates of OS are shown in Figure 4 up to 10 years.

**Figure 4.**
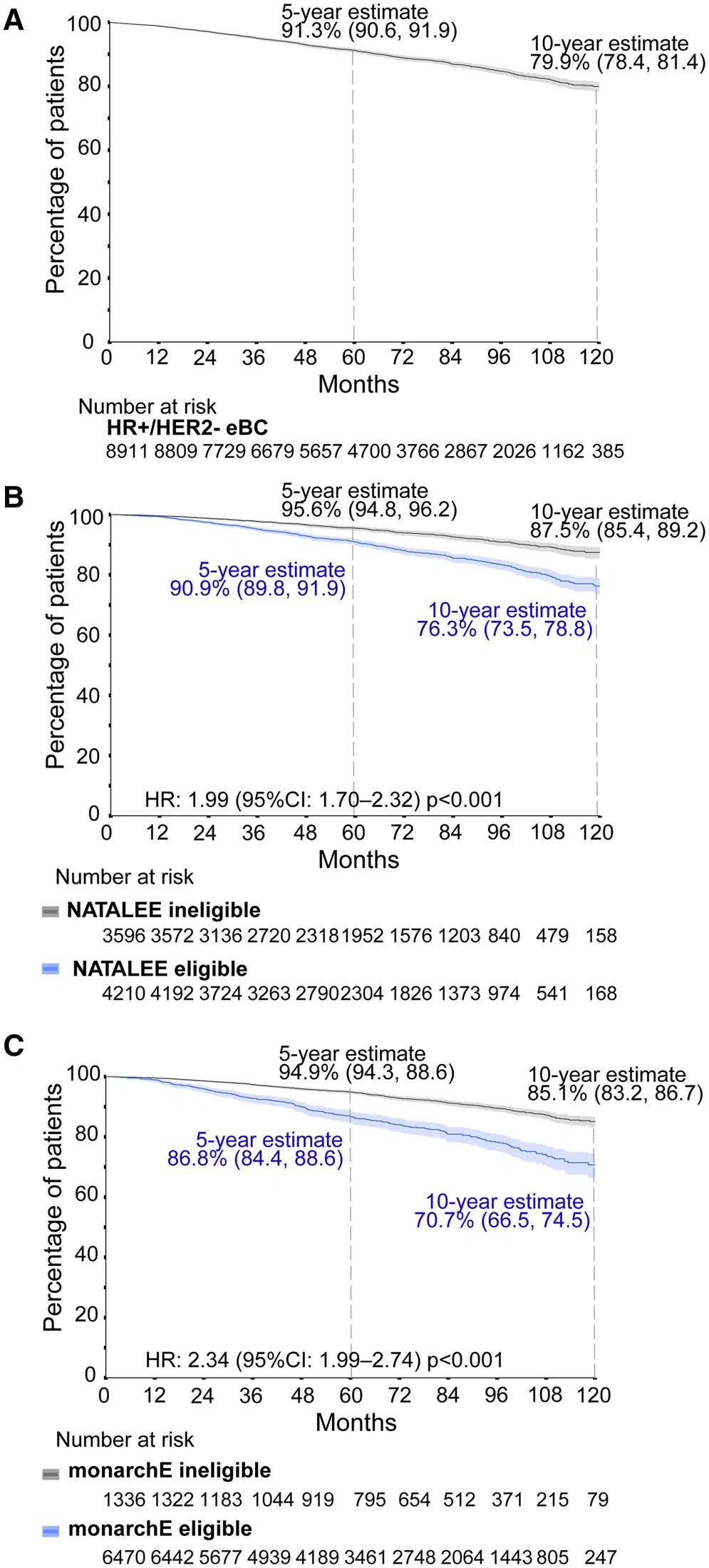
Kaplan-Meier estimates for overall survival (OS) among patients with HR+/HER2− eBC (A) overall, (B) per NATALEE eligibility (eligible vs ineligible, and (C) per monarchE eligibility criteria (eligible vs ineligible).

Mortality risk was higher among eligible patients compared with ineligible patients (NATALEE-eligible: HR 1.94; 95% CI 1.66-2.28; *P* < .001; monarchE-eligible: HR 2.35; 95% CI 2.00-2.76; *P* < .001; Figure S1c, see online supplementary material for a color version of this figure).

### Exploratory analyses: iDFS, DDFS, and OS by nodal status

Exploratory outcome analyses according to nodal status were conducted for iDFS, DDFS, and OS among NATALEE-eligible and monarchE-eligible patients (Figures S2-S4, see online supplementary material for a color version of these figures). Of the 5149 patients with N0 disease, 615 fulfilled the NATALEE high-risk N0 eligibility criteria. Outcomes in this subgroup were comparable to, and numerically slightly worse than, those observed among NATALEE-eligible patients with N1 disease. Five-year iDFS, DDFS, and OS estimates were 83.1% (95% CI 79.5-86.1), 89.6% (95% CI 86.5-92.0), and 91.3% (95% CI 88.4-93.5), respectively, among NATALEE-eligible N0 patients, compared with 84.6% (95% CI 82.9-86.2), 90.4% (95% CI 88.9-91.6), and 92.5% (95% CI 91.2-93.6) among NATALEE-eligible N1 patients. At 10 years, iDFS, DDFS, and OS remained numerically lower in NATALEE-eligible N0 than N1 patients (63.6% vs 72.3%, 71.5% vs 79.2%, and 77.4% vs 80.2%, respectively). These estimates should be interpreted with caution because of the limited number of patients remaining at risk.

Among monarchE-eligible patients, those with N2-N3 disease had less favorable outcomes than patients with N1 disease. Five-year iDFS, DDFS, and OS estimates for patients with N2-N3 disease were 74.4% (95% CI 70.5-77.9), 80.1% (95% CI 76.4-83.2), and 85.3% (95% CI 82.0-88.1), respectively. Corresponding 5-year estimates for N1-patient population were 79.0% (95% CI 75.5-82.0), 84.3% (95% CI 81.1-86.9), and 88.1% (95% CI 85.2-90.4), respectively. Detailed Kaplan–Meier estimates for all nodal subgroups are presented in Figures S2-S4 (see online supplementary material for a color version of these figures).

## Discussion

In this population-based cohort study, we evaluated the real‑world proportion and long-term outcomes of patients with HR+/HER2- eBC who would be candidates for adjuvant CDK4/6 inhibitors according to the pathological and anatomical eligibility criteria applied in the NATALEE and monarchE trials. Approximately 40% of all HR+/HER2– eBC patients (46% among those with sufficient data to determine eligibility) met the NATALEE criteria and 15% (17% among those with sufficient data to determine eligibility) the monarchE criteria, highlighting the substantial proportion of patients potentially targeted by adjuvant CDK4/6 inhibition in routine clinical practice. Our estimates for the proportion of NATALEE-eligible patients are broadly consistent with previous real-world reports, including Swedish and German cohorts (38% and 43%, respectively),^19^^,^^27^ and fall between those reported in Italy (56%)^28^ and the United States (31%),^29^ despite differences in study methods.

Eligible patients demonstrated substantially poorer long‑term outcomes than ineligible patients, consistent with the 5-year outcomes reported in the Results. Longer-term Kaplan–Meier estimates suggested that this excess risk persisted over time: at 10 years, iDFS, DDFS, and OS were 66%, 74%, and 76% for NATALEE‑eligible patients, and 60%, 67%, and 71% for monarchE‑eligible patients, respectively. At this time point, more than one third of eligible patients in both groups had experienced disease recurrence, predominantly at distant sites. In line with these time‑to‑event analyses, progression to aBC was substantially more frequent among eligible patients: during follow‑up, 6.5% of NATALEE-eligible patients and 12.5% of monarchE-eligible patients developed advanced disease, with significantly higher proportions compared with their respective ineligible cohorts. As distant metastatic progression constitutes a defining event for DDFS and contributes to iDFS, the higher frequency of progression to aBC among eligible patients is consistent with the observed differences in long‑term survival outcomes. Eligible patients also differed in baseline characteristics, notably by younger age, which has been consistently associated with higher proliferation and more aggressive HR+/HER2– tumor biology.^10^^,^^11^^,^^30–32^ These features likely contribute to the limited ability of standard ET ± chemotherapy to fully mitigate recurrence risk in this population^10^^,^^11^^,^^14^^,^^33^ and support the rationale for adjuvant CDK4/6 inhibition.^17^^,^^18^ Reflecting this adverse biology, eligible patients experienced substantially higher rates of distant metastatic recurrence, consistent with the observed differences in DDFS and underscoring the importance of accurate early risk identification and timely therapeutic intensification.

Overall, outcome patterns were broadly consistent with those reported in the respective phase III trials^15^^,^^18^ despite differences in underlying clinical characteristics, stage distribution, and real-world patient heterogeneity. Notably, the 5-year iDFS observed in our study for both eligible cohorts (NATALEE 83% and monarchE 77%) was numerically comparable to that reported in the corresponding trials (NATALEE 81% and monarchE 77%).^15^^,^^18^ Nordic real-world studies have similarly reported 5-year recurrence-free or iDFS estimates of approximately 75%-83% for NATALEE-eligible populations and 70%-77% for monarchE eligible populations over 4-7 years of follow-up.^19–21^ Together, these findings support the applicability of trial-defined eligibility criteria in routine practice and underscore the substantial residual risk of invasive recurrence observed among NATALEE- and monarchE-eligible patients treated with standard adjuvant endocrine therapy. Exploratory analyses according to nodal status demonstrated heterogeneity in outcomes across nodal subgroups. Importantly, patients with high-risk N0 disease meeting the NATALEE criteria experienced outcomes comparable to, and in some analyses numerically slightly worse than, those observed among N1 patients, indicating that nodal negativity alone should not be interpreted as low risk when additional high-risk clinicopathologic features are present. Key strengths of this study include its population-based design, comprehensive electronic health record data, and long duration of follow‑up. Finland’s unified healthcare structure enabled near complete capture of HR+/HER2– eBC cases within a well-defined regional cohort covering approximately 30% (1.6 of 5.6 million) of the national population, supporting the generalizability of findings to similar healthcare settings. The use of both structured data and free‑text clinical information allowed robust ascertainment of recurrences and metastatic progression. Several limitations inherent to real-world datasets should be acknowledged, including incomplete Ki‑67 reporting, missing pathological variables, lack of genomic profiling data, and potential misclassification arising from registry-based definitions. Importantly, genomic risk assessment could not be incorporated into the NATALEE eligibility algorithm because genomic test results were not available in the data source and were only infrequently used in Finnish clinical practice during most of the study period. Consequently, some patients who may have fulfilled the genomic-risk criterion of the NATALEE trial could have been classified as ineligible in our study, potentially leading to an underestimation of NATALEE eligibility. These factors may introduce some uncertainty but are unlikely to affect the overall risk patterns observed. Eligibility in the NATALEE and monarchE trials also incorporated clinical criteria beyond pathological and anatomical factors, such as prior cancers and performance status, which could not be fully assessed in this study; therefore, trial‑eligible populations are not completely overlapping with those identified in our real‑world cohort. In addition, the study was designed to independently replicate and evaluate the NATALEE and monarchE eligibility frameworks in a real‑world setting rather than compare overlapping and non‑overlapping eligibility groups directly. Consequently, outcomes among patients who fulfilled only NATALEE criteria were not analyzed separately from those meeting monarchE criteria, representing a potential area for future research. Finally, treatment patterns reflect clinical practice from a period preceding the widespread adoption of CDK4/6 inhibitors, thereby providing an estimate of baseline prognosis. As these agents become increasingly incorporated into routine clinical practice, real world prognosis is expected to improve.

In conclusion, our findings demonstrate that the trial‑defined NATALEE and monarchE eligibility criteria identify a substantial proportion of real‑world HR+/HER2- eBC patients who face markedly elevated long-term risks of recurrence and mortality. Furthermore, these results provide robust real-world estimates of baseline prognosis in trial-eligible populations prior to the implementation of adjuvant CDK4/6 inhibitors, confirm the prognostic relevance of these risk frameworks in routine clinical practice, and provide important context for the interpretation and implementation of adjuvant CDK4/6 inhibition.

## Supplementary Material

oyag419_Supplementary_Data

## Data Availability

This study is based on secondary use of social and healthcare registry data. Thus, data cannot be shared (per Finnish legislation) without a separate study extension permission process. Data can be acquired with data permission by following the guidance and application process of the registry.
