# Supplementary material for: Characteristics and outcomes of NATALEE- and monarchE-eligible early breast cancer in Finland: a regional real-world cohort study
Source: Oncologist. 2026 Oct 20;31(12):oyag419. doi: 10.1093/oncolo/oyag419 (PMC13755885; doi:10.1093/oncolo/oyag419)
Supplement: Supplementary file 1 [file oyag419_supplementary_data.docx]

Table S1. Inclusion to at risk of recurrence cohorts per NATALEE^13^ and monarchE^14^ cohort 1 criteria. Staging was performed according to the anatomic classification in the AJCC Cancer Staging Manual, 8th edition.

| **AJCC Anatomical Staging** | **Tumor size (T)** | **Nodal status (N)** | **NATALEE** | **monarchE cohort 1** |
| --- | --- | --- | --- | --- |
| **Stage IIA** | **T0-1** | **N1** | yes | Only if grade 3 |
|  | **T2** | **N0** | Only if G3; or G2 with Ki-67 ≥20% or high genomic risk* | no |
| **Stage IIB** | **T2** | **N1** | yes | Only if grade 3 |
|  | **T3** | **N0** | yes | no |
| **Stage IIIA** | **T0-2** | **N2** | yes | yes |
|  | **T3** | **N1-2** | yes | yes |
| **Stage IIIB** | **T4** | **N0** | yes | no |
|  | **T4** | **N1** | yes | Only if tumor size ≥5 cm or grade 3 |
|  | **T4** | **N2** | yes | yes |
| **Stage IIIC** | **Any T** | **N3** | yes | yes |

**High genomic risk was not assessed in this study due to lack of appropriate data.*

**Table S2.** Baseline characteristics, female patients with HR+/HER2- eBC according to menopause status and NATALEE/monarchE eligibility.

| **Characteristic** | **NATALEE eligible, post-menopausal,  N = 2,498** | **NATALEE eligible, pre-menopausal,  N = 1,078** | **NATALEE ineligible, post-menopausal,  N = 3,362** | **NATALEE ineligible, pre-menopausal,  N = 838** | **monarchE eligible, postmenopausal  N = 847** | **monarchE eligible, premenopausal  N = 478** | **monarchE ineligible, postmenopausal  N = 5,013** | **monarchE ineligible, premenopausal  N = 1,438** |
| --- | --- | --- | --- | --- | --- | --- | --- | --- |
| **Age at diagnosis, years, median (IQI)** | 69.8 (64.2-76.6) | 50.5 (46.4-55.1) | 68.2 (62.8-74.2) | 51.0 (47.7-54.5) | 70.2 (64.7-76.7) | 50.6 (46.2-55.2) | 68.6 (62.9-74.9) | 50.8 (47.1-54.7) |
| **Age at diagnosis, categories** |  |  |  |  |  |  |  |  |
| **Less than 40** | 8 (0.3%) | 74 (6.9%) | 8 (0.2%) | 49 (5.8%) | 4 (0.5%) | 33 (6.9%) | 12 (0.2%) | 90 (6.3%) |
| **40 - 49** | 10 (0.4%) | 404 (37.5%) | 7 (0.2%) | 272 (32.5%) | 3 (0.4%) | 180 (37.7%) | 14 (0.3%) | 496 (34.5%) |
| **50 - 59** | 211 (8.4%) | 600 (55.7%) | 399 (11.9%) | 517 (61.7%) | 54 (6.4%) | 265 (55.4%) | 556 (11.1%) | 852 (59.2%) |
| **60 - 69** | 1,034 (41.4%) | 0 | 1,641 (48.8%) | 0 | 360 (42.5%) | 0 | 2,315 (46.2%) | 0 |
| **70 - 79** | 847 (33.9%) | 0 | 993 (29.5%) | 0 | 291 (34.4%) | 0 | 1,549 (30.9%) | 0 |
| **80+** | 388 (15.5%) | 0 | 314 (9.3%) | 0 | 135 (15.9%) | 0 | 567 (11.3%) | 0 |
| **Sex** |  |  |  |  |  |  |  |  |
| **Female** | 2,498 (100%) | 1,078 (100%) | 3,362 (100%) | 838 (100%) | 847 (100%) | 478 (100%) | 5,013 (100%) | 1,438 (100%) |
| **NATALEE** |  |  |  |  |  |  |  |  |
| **Eligible** | 2,498 (100%) | 1,078 (100%) | 0 | 0 | 847 (100%) | 478 (100%) | 1,651 (32.9%) | 600 (41.7%) |
| **Ineligible** | 0 | 0 | 3,362 (100%) | 838 (100%) | 0 | 0 | 3,362 (67.1%) | 838 (58.3%) |
| **monarchE** |  |  |  |  |  |  |  |  |
| **Eligible** | 847 (33.9%) | 478 (44.3%) | 0 | 0 | 847 (100%) | 478 (100%) | 0 | 0 |
| **Ineligible** | 1,651 (66.1%) | 600 (55.7%) | 3,362 (100%) | 838 (100%) | 0 | 0 | 5,013 (100%) | 1,438 (100%) |
| **Metastatic axillary lymph nodes** | 2,064 (82.6%) | 901 (83.6%) | 0 | 0 | 847 (100%) | 478 (100%) | 1,217 (24.3%) | 423 (29.4%) |
| **Number of metastatic lymph nodes** |  |  |  |  |  |  |  |  |
| **0** | 434 (17.4%) | 177 (16.4%) | 3,362 (100%) | 838 (100%) | 0 | 0 | 3,796 (75.7%) | 1,015 (70.6%) |
| **1-3** | 1,672 (66.9%) | 695 (64.5%) | 0 | 0 | 455 (53.7%) | 272 (56.9%) | 1,217 (24.3%) | 423 (29.4%) |
| **4-9** | 201 (8.0%) | 124 (11.5%) | 0 | 0 | 201 (23.7%) | 124 (25.9%) | 0 | 0 |
| **> 9** | 191 (7.6%) | 82 (7.6%) | 0 | 0 | 191 (22.6%) | 82 (17.2%) | 0 | 0 |
| **Grade** |  |  |  |  |  |  |  |  |
| **1** | 352 (14.9%) | 138 (13.3%) | 1,319 (41.6%) | 325 (41.4%) | 43 (5.2%) | 23 (4.9%) | 1,628 (34.6%) | 440 (32.6%) |
| **2** | 1,260 (53.4%) | 472 (45.6%) | 1,495 (47.2%) | 347 (44.2%) | 226 (27.5%) | 121 (25.7%) | 2,529 (53.7%) | 698 (51.7%) |
| **3** | 749 (31.7%) | 426 (41.1%) | 355 (11.2%) | 113 (14.4%) | 553 (67.3%) | 326 (69.4%) | 551 (11.7%) | 213 (15.8%) |
| **Unknown** | 137 (5.5%) | 42 (3.9%) | 193 (5.7%) | 53 (6.3%) | 25 (3.0%) | 8 (1.7%) | 305 (6.1%) | 87 (6.1%) |
| **Tumor size** |  |  |  |  |  |  |  |  |
| **<= 2 cm** | 1,077 (43.1%) | 390 (36.2%) | 3,050 (90.7%) | 770 (91.9%) | 298 (35.2%) | 150 (31.4%) | 3,829 (76.4%) | 1,010 (70.2%) |
| **2 - 5 cm** | 1,206 (48.3%) | 551 (51.1%) | 312 (9.3%) | 68 (8.1%) | 400 (47.2%) | 221 (46.2%) | 1,118 (22.3%) | 398 (27.7%) |
| **> 5 cm** | 209 (8.4%) | 137 (12.7%) | 0 | 0 | 146 (17.2%) | 107 (22.4%) | 63 (1.3%) | 30 (2.1%) |
| **Spred to surrounding**  **tissue** | 6 (0.2%) | 0 | 0 | 0 | 3 (0.4%) | 0 | 3 (0.1%) | 0 |
| **Ki-67 expression** |  |  |  |  |  |  |  |  |
| **< 20%** | 926 (37.9%) | 319 (30.4%) | 2,369 (72.1%) | 536 (67.1%) | 185 (22.4%) | 81 (17.3%) | 3,110 (63.5%) | 774 (56.0%) |
| **>= 20%** | 1,516 (62.1%) | 732 (69.6%) | 915 (27.9%) | 263 (32.9%) | 640 (77.6%) | 386 (82.7%) | 1,791 (36.5%) | 609 (44.0%) |
| **Unknown** | 56 (2.2%) | 27 (2.5%) | 78 (2.3%) | 39 (4.7%) | 22 (2.6%) | 11 (2.3%) | 112 (2.2%) | 55 (3.8%) |
| **Developed aBC during follow-up** | 160 (6.4%) | 70 (6.5%) | 53 (1.6%) | 18 (2.1%) | 109 (12.9%) | 55 (11.5%) | 104 (2.1%) | 33 (2.3%) |
| **CCI** |  |  |  |  |  |  |  |  |
| **0** | 2,079 (83.2%) | 1,010 (93.7%) | 2,861 (85.1%) | 779 (93.0%) | 699 (82.5%) | 446 (93.3%) | 4,241 (84.6%) | 1,343 (93.4%) |
| **1** | 147 (5.9%) | 28 (2.6%) | 208 (6.2%) | 29 (3.5%) | 46 (5.4%) | 14 (2.9%) | 309 (6.2%) | 43 (3.0%) |
| **2+** | 272 (10.9%) | 40 (3.7%) | 293 (8.7%) | 30 (3.6%) | 102 (12.0%) | 18 (3.8%) | 463 (9.2%) | 52 (3.6%) |
| **BC diagnosis year** |  |  |  |  |  |  |  |  |
| **2014 - 2016** | 783 (31.3%) | 413 (38.3%) | 1,016 (30.2%) | 252 (30.1%) | 334 (39.4%) | 205 (42.9%) | 1,465 (29.2%) | 460 (32.0%) |
| **2017 - 2019** | 757 (30.3%) | 352 (32.7%) | 1,091 (32.5%) | 285 (34.0%) | 264 (31.2%) | 150 (31.4%) | 1,584 (31.6%) | 487 (33.9%) |
| **2020 - 2022** | 809 (32.4%) | 261 (24.2%) | 1,088 (32.4%) | 265 (31.6%) | 213 (25.1%) | 103 (21.5%) | 1,684 (33.6%) | 423 (29.4%) |
| **2023 - 2024** | 149 (6.0%) | 52 (4.8%) | 167 (5.0%) | 36 (4.3%) | 36 (4.3%) | 20 (4.2%) | 280 (5.6%) | 68 (4.7%) |
| **Histology** |  |  |  |  |  |  |  |  |
| **Carcinoma ductale** | 1,696 (68.8%) | 797 (74.8%) | 2,311 (69.9%) | 641 (77.1%) | 598 (72.0%) | 355 (75.9%) | 3,409 (69.0%) | 1,083 (75.8%) |
| **Carcinoma lobular** | 593 (24.1%) | 218 (20.5%) | 648 (19.6%) | 127 (15.3%) | 185 (22.3%) | 93 (19.9%) | 1,056 (21.4%) | 252 (17.6%) |
| **Carcinoma mucinosum** | 41 (1.7%) | 3 (0.3%) | 104 (3.1%) | 11 (1.3%) | 7 (0.8%) | < 3 | 138 (2.8%) | 13 (0.9%) |
| **Carcinoma**  **neuroendocrinum** | 15 (0.6%) | < 3 | 9 (0.3%) | 0 | < 3 | 0 | 22 (0.4%) | < 3 |
| **Carcinoma tubulare** | 9 (0.4%) | 6 (0.6%) | 85 (2.6%) | 27 (3.2%) | < 3 | 0 | 93 (1.9%) | 33 (2.3%) |
| **Multiple histologies** | 106 (4.3%) | 39 (3.7%) | 135 (4.1%) | 25 (3.0%) | 34 (4.1%) | censored | 207 (4.2%) | 45 (3.2%) |
| **Other*** | 5 (0.2%) | < 3 | 13 (0.4%) | 0 | 3 (0.4%) | 0 | 15 (0.3%) | < 3 |
| **Unknown** | 33 (1.3%) | 13 (1.2%) | 57 (1.7%) | 7 (0.8%) | 17 (2.0%) | 10 (2.1%) | 73 (1.5%) | 10 (0.7%) |
| **Length of follow-up, total (from BC dx until EOF), median (IQI)** | 5.1 (2.8-7.5) | 6.2 (3.8-8.3) | 5.3 (3.2-7.7) | 5.7 (3.4-8.0) | 5.6 (3.0-8.1) | 6.6 (4.1-8.4) | 5.1 (3.0-7.6) | 5.8 (3.5-8.1) |
| **Length of follow-up, early (from BC dx until EOF or occurrence of aBC), median (IQI)** | 4.8 (2.7-7.4) | 6.1 (3.6-8.2) | 5.3 (3.2-7.7) | 5.6 (3.3-7.8) | 5.3 (2.7-7.8) | 6.3 (3.8-8.4) | 5.1 (3.0-7.6) | 5.8 (3.4-8.0) |

*Abbreviations: eBC, early breast cancer; aBC, advanced breast cancer; IQI, interquartile range.*

*Data are shown as n (%) unless otherwise indicated.*

*Due to anonymity guidelines by the Finnish Central Authority Findata, small patient groups (<3 patients) have not been reported in detail. Additionally, numbers in other cells have been censored, if reporting would have allowed inferring the exact number covered by “<3”.*

**Histological subtype one of: ca. apocrinum, ca. metaplasticum, ca. papillare, ca. medullare mammae, or ca. adenomatosum****.***

**Table S3.** Nodal Status and Tumor Size of NATALEE eligible (a) and ineligible (b) and monarchE eligible (c) and ineligible (d) patients. Patients classified as T4 were not included due to low number of patients (NATALEE and monarchE eligible n=3).

a)

| **NATALEE eligible** | | **Nodal Status (N)** | | | | |
| --- | --- | --- | --- | --- | --- | --- |
|  |  | **N0** | **N1** | **N2** | **N3** | **Any** |
| **Tumor size (T)** | **T1** | NA | Stage IIA (T1N1)  n=1270 (35.4%) | Stage IIIA (T1N2)  n=101 (2.8%) | Stage IIIC (T1N3)  n=101 (2.8%) | n=1472 (41.0%) |
|  | **T2** | Stage IIA (T2N0)  n=519 (14.5%) | Stage IIB (T2N1)  n=964 (26.9%) | Stage IIIA (T2N2)  n=175 (4.9%) | Stage IIIC (T2N3)  n=114 (3.2%) | n=1772 (49.4%) |
|  | **T3** | Stage IIB (T3N0)  n=93 (2.6%) | Stage IIIA (T3N1)  n=142 (4.0%) | Stage IIIA (T3N2)  n=51 (1.4%) | Stage IIIC (T3N3)  n=60 (1.7%) | n=346 (9.6%) |
|  | **Any** | n=612 (17.1%) | n=2376 (66.2%) | n=327 (9.1%) | n=275 (7.7%) | n=3590 (100%) |

b)

| **NATALEE ineligible** | | **N0** |
| --- | --- | --- |
| **Tumor size (T)** | **T1** | Stage I (T1N0)  n=3830 (9.0%) |
|  | **T2** | Stage IIA (T2N0)  n=380 (9.0%) |
|  | **Any** | n=4210 (100%) |

c)

| **monarchE eligible** | | **Nodal Status (N)** | | | |
| --- | --- | --- | --- | --- | --- |
|  |  | **N1** | **N2** | **N3** | **Any** |
| **Tumor size (T)** | **T1** | Stage IIA (T1N1)  n=247 (18.5%) | Stage IIIA (T1N2)  n=101 (7.6%) | Stage IIIC (T1N3)  n=101 (7.6%) | n=449 (33.7%) |
|  | **T2** | Stage IIB (T2N1)  n=342 (25.7%) | Stage IIIA (T2N2)  n=175 (13.1%) | Stage IIIC (T2N3)  n=114 (8.6%) | n=631 (47.3%) |
|  | **T3** | Stage IIIA (T3N1)  n=142 (10.7%) | Stage IIIA (T3N2)  n=51 (3.8%) | Stage IIIC (T3N3)  n=60 (4.5%) | n=253 (19.0%) |
|  | **Any** | n=731 (54.8%) | n=327 (24.5%) | n=275 (20.6%) | n=1333 (100%) |

d)

| **monarchE ineligible** | | **Nodal Status (N)** | | |
| --- | --- | --- | --- | --- |
|  |  | **N0** | **N1** | **Any** |
| **Tumor size (T)** | **T1** | Stage I (T1N0)  n=3830 (59.2%) | Stage IIA (T1N1)  n=1023 (15.8%) | n=4853 (75.0%) |
|  | **T2** | Stage IIA (T2N0)  n=899 (13.9%) | Stage IIB (T2N1)  n=622 (9.6%) | n=1521 (23.5%) |
|  | **T3** | Stage IIB (T3N0)  n=93 (1.4%) | NA | n=93 (1.4%) |
|  | **Any** | n=4822 (74.6%) | n=1645 (25.4%) | n=6467 (100%) |

**Supplemental Figure S1.** Multivariable cause specific Cox models to assess a) iDFS (event defined as recurrence or distant metastasis; patients censored at death, secondary malignancy, or end of follow-up), b) DDFS (event defined as distant metastasis; secondary malignancy, patients censored at death or end of follow-up), and c) OS association with NATALEE and monarchE eligibility statuses while adjusting for sex, age category at diagnosis, CCI, year of breast cancer diagnosis, menopausal status, and histology. The hazard ratios (HR), 95% confidence intervals (CI), and p-values are reported.

a) iDFS

**“Other” histology includes subtypes of apocrinum/metaplasticum/papillare/medullare mammae/adenomatosum for OS and aforementioned combined with mucinosum, neuroendocrinum, and tubulare.*

b) DDFS

**“Other” histology includes subtypes of apocrinum/metaplasticum/papillare/medullare mammae/adenomatosum for OS and aforementioned combined with mucinosum, neuroendocrinum, and tubulare*.

*Due to anonymity guidelines by the Finnish Central Authority Findata, small patient groups (<3 patients) have not been reported in detail. Additionally, numbers in other cells have been censored, if reporting would have allowed inferring the exact number covered by “<3”.*

c) OS

**“Other” histology includes subtypes of apocrinum/metaplasticum/papillare/medullare mammae/adenomatosum for OS and aforementioned combined with mucinosum, neuroendocrinum, and tubulare.*

*Due to anonymity guidelines by the Finnish Central Authority Findata, small patient groups (<3 patients) have not been reported in detail. Additionally, numbers in other cells have been censored, if reporting would have allowed inferring the exact number covered by “<3”.*

*Abbreviations: T, Tumor size; N, Nodal status.*

**Supplemental Figure S2.** Kaplan–Meier estimates of invasive disease-free survival (iDFS) according to nodal status among eligible patients with HR+/HER2− early breast cancer: (A) NATALEE-eligible patients stratified by node-negative disease (N0), 1–3 positive axillary lymph nodes (N1), and ≥4 positive axillary lymph nodes (N2–N3); and (B) monarchE-eligible patients stratified by N1 and N2–N3 disease.

**Supplemental Figure S3.** Kaplan–Meier estimates of distant disease-free survival (DDFS) according to nodal status among eligible patients with HR+/HER2− early breast cancer: (A) NATALEE-eligible patients stratified by node-negative disease (N0), 1–3 positive axillary lymph nodes (N1), and ≥4 positive axillary lymph nodes (N2–N3); and (B) monarchE-eligible patients stratified by N1 and N2–N3 disease. Five- and ten-year DDFS estimates are shown for each nodal subgroup.

**Supplemental Figure S4.** Kaplan–Meier estimates of overall survival (OS) according to nodal status among eligible patients with HR+/HER2− early breast cancer: (A) NATALEE-eligible patients stratified by node-negative disease (N0), 1–3 positive axillary lymph nodes (N1), and ≥4 positive axillary lymph nodes (N2–N3); and (B) monarchE-eligible patients stratified by N1 and N2–N3 disease. Five- and ten-year OS estimates are shown for each nodal subgroup.

**Table S4.** Treatment patterns by eligibility criteria a) and more detailed treatment patterns further stratified by menopausal status b).

a)

| **Timing of treatment** | **Treatment type** | **All HR+/HER2- eBC patients N = 8,911** | | **NATALEE** | | | | **monarchE** | | | |
| --- | --- | --- | --- | --- | --- | --- | --- | --- | --- | --- | --- |
|  |  |  |  | **Eligible  N = 3,582*** | | **Ineligible  N = 4,198*** | | **Eligible  N = 1,327*** | | **Ineligible  N = 6,453*** | |
|  |  | **N** | **%** | **N** | **%** | **N** | **%** | **N** | **%** | **N** | **%** |
| **Partial or complete mastectomy** | | 8637 | 96.9 | 3582 | 100 | 4198 | 100 | 1327 | 100 | 6453 | 100 |
| **Neoadjuvant** | Chemotherapy | 314 | 3.5 | 71 | 2.0 | 67 | 1.6 | 23 | 1.7 | 115 | 1.8 |
|  | Endocrine therapy | 219 | 2.5 | 61 | 1.7 | 43 | 1.0 | 38 | 2.9 | 66 | 1.0 |
|  | GnRH | 33 | 0.4 | 9 | 0.3 | <3 | - | 6 | 0.5 | 4 | 0.1 |
| **Adjuvant** | Chemotherapy | 2008 | 22.5 | 1628 | 45.4 | 224 | 5.3 | 743 | 56.0 | 1109 | 17.2 |
|  | Endocrine therapy | 7391 | 82.9 | 3484 | 97.3 | 2942 | 70.1 | 1298 | 97.8 | 5128 | 79.5 |
|  | GnRH | 324 | 3.6 | 184 | 5.1 | 71 | 1.7 | 89 | 6.7 | 166 | 2.6 |

**Patients with partial or complete mastectomy.*

b)

|  | | **All female patients with HR+/HER2- eBC** | | | | **NATALEE-eligible** | | | | **NATALEE-ineligible** | | | | **monarchE-eligible** | | | | **monarchE-ineligible** | | | |
| --- | --- | --- | --- | --- | --- | --- | --- | --- | --- | --- | --- | --- | --- | --- | --- | --- | --- | --- | --- | --- | --- |
|  |  | **Post-menopausal** | | **Pre-menopausal** | | **Post-menopausal** | | **Pre-menopausal** | | **Post-menopausal** | | **Pre-menopausal** | | **Post-menopausal** | | **Pre-menopausal** | | **Post-menopausal** | | **Pre-menopausal** | |
|  |  |  | |  | | **N=2484*** | | **N=1078*** | | **N=3354*** | | **N=834*** | | **N=838*** | | **N=478*** | | **N=5000*** | | **N=1434*** | |
| **Timing of treatment** | **Treatment type** | **N** | **%** | **N** | **%** | **N** | **%** | **N** | **%** | **N** | **%** | **N** | **%** | **N** | **%** | **N** | **%** | **N** | **%** | **N** | **%** |
| **Partial or complete mastectomy** | | 6466 | 96.2 | 2137 | 99.2 | 2484 | 100.0 | 1078 | 100.0 | 3354 | 100.0 | 834 | 100.0 | 838 | 100.0 | 478 | 100.0 | 5000 | 100.0 | 1434 | 100.0 |
| **Neoadjuvant** | Chemotherapy | 187 | 2.8 | 125 | 5.8 | 51 | 2.1 | 19 | 1.8 | 59 | 1.8 | 8 | 1.0 | 12 | 1.4 | 11 | 2.3 | 98 | 2.0 | 16 | 1.1 |
|  | Endocrine therapy | 143 | 2.1 | 74 | 3.4 | 38 | 1.5 | 22 | 2.0 | 36 | 1.1 | 7 | 0.8 | 20 | 2.4 | 17 | 3.6 | 54 | 1.1 | 12 | 0.8 |
|  | GnRH | 3 | 0.0 | 29 | 1.3 | <3 | - | 8 | 0.7 | 0 | 0.0 | <3 | - | <3 | - | 5 | 1.0 | 0 | 0.0 | 4 | 0.3 |
| **Adjuvant** | Chemotherapy | 946 | 14.1 | 1055 | 49.0 | 788 | 31.7 | 833 | 77.3 | 82 | 2.4 | 142 | 17.0 | 361 | 43.1 | 379 | 79.3 | 509 | 10.2 | 596 | 41.6 |
|  | Endocrine therapy | 5588 | 83.1 | 1771 | 82.2 | 2413 | 97.1 | 1051 | 97.5 | 2417 | 72.1 | 515 | 61.8 | 814 | 97.1 | 473 | 99.0 | 4016 | 80.3 | 1093 | 76.2 |
|  | GnRH | 35 | 0.5 | 284 | 13.2 | 13 | 0.5 | 170 | 15.8 | 11 | 0.3 | 57 | 6.8 | 6 | 0.7 | 82 | 17.2 | 18 | 0.4 | 145 | 10.1 |

**Patients with surgery of the primary tumor; GnRH=Gonadotropin Releasing Hormone.*
